# Custom, spray coated receive coils for magnetic resonance imaging

**DOI:** 10.1038/s41598-021-81833-0

**Published:** 2021-01-29

**Authors:** A. M. Zamarayeva, K. Gopalan, J. R. Corea, M. Z. Liu, K. Pang, M. Lustig, A. C. Arias

**Affiliations:** Department of Electrical and Computer Engineering, University of California Berkeley, Berkeley, CA USA

**Keywords:** Electrical and electronic engineering, Magnetic resonance imaging, Electronic devices, Sensors and biosensors

## Abstract

We have developed a process for fabricating patient specific Magnetic Resonance Imaging (MRI) Radio-frequency (RF) receive coil arrays using additive manufacturing. Our process involves spray deposition of silver nanoparticle inks and dielectric materials onto 3D printed substrates to form high-quality resonant circuits. In this paper, we describe the material selection and characterization, process optimization, and design and testing of a prototype 4-channel neck array for carotid imaging. We show that sprayed polystyrene can form a low loss dielectric layer in a parallel plate capacitor. We also demonstrate that by using sprayed silver nanoparticle ink as conductive traces, our devices are still dominated by sample noise, rather than material losses. These results are critical for maintaining high Signal-to-Noise-Ratio (SNR) in clinical settings. Finally, our prototype patient specific coil array exhibits higher SNR (5 × in the periphery, 1.4 × in the center) than a commercially available array designed to fit the majority of subjects when tested on our custom neck phantom. 3D printed substrates ensure an optimum fit to complex body parts, improve diagnostic image quality, and enable reproducible placement on subjects.

## Introduction

Healthcare technology can be significantly improved through customization to individual patients^1–3^. Magnetic resonance imaging (MRI) is one of the examples where the customization of hardware could appreciably advance clinical outcomes. One of the key factors determining signal-to-noise ratio (SNR) of MR images is the design of the receive coils that are used to collect RF signal and their proximity to the patient^6–8^. Particularly, placing coils close to the body has been shown to significantly improve SNR and, thus, diagnostic image quality^9,10^. However, commercially available receive coils are typically designed to accommodate the largest possible subjects and do not optimally fit every patient or subject. This often results in substantial gaps between the coils and the body, which in turn compromises SNR. For instance, Corea et al. showed that placing coil only 1.8 cm away from the body results in an ~ 8% decrease in SNR^9^. Additionally, conventional coils are not designed for reproducible positioning on the patient, and do not restrict a patient from moving, which leads to motion artifacts during MRI scans. These shortcomings hinder the development of the next generation therapeutic approaches, such as MRI guided surgeries^11–13^, that require multiple time-consuming MRI scans on a given patient visit. In addition, fMRI researchers who perform repeated scans on the same subject may find the reproducible placement of the coils beneficial.

Custom receive coils that are fabricated on-demand to fit a patient’s or subject’s anatomy would address some of the aforementioned limitations. However, the established commercial manufacturing process is not suitable for on-demand and custom coil production. Typical coil manufacturing requires a trained RF engineer and involves hand assembly and packaging of electronic components such as copper wires and porcelain capacitors^14^. Entirely new approaches that allow seamless manufacturing and integration of electronic elements must be adopted to enable custom MRI coils. Novel additive manufacturing techniques and solution-processed materials offer a potential to transform patient-specific coil manufacturing^15,16^. The common concern with shifting towards solution-processed electronic materials is the higher loss associated with their use; for example, printed solution-based conductors exhibit lower conductivities than that of bulk metals^17^. However, in clinical MRI intrinsic losses in the system are dominated by losses stemming from the human body^18,19^. Therefore, printed materials could perform comparably or better than the conventional materials, while enabling additive manufacturing of custom coils.

The first demonstrations of the conformal MRI receive coils did not rely on additive manufacturing and were fabricated by sewing conductors into fabric^20^, using mercury^21,22^, or copper tape^23–25^ as a conductor. Mager et al. produced flexible coils using ink-jet printing^26^. While inkjet printing allows printing coils onto the flexible substrates, it requires many printing passes to achieve the desired conductivity for RF applications^15^. Corea et al. developed highly flexible and lightweight receive coils that were fabricated using scalable and low-cost screen-printing approach^9,10,13^. This paved the way to new opportunities in imaging, particularly for pediatric patients for whom conventional adult coils are especially problematic.

In this work we developed a process for additive manufacturing of 3D patient-specific MRI coils. Such coils are advantageous for applications where, in addition to improved SNR and reproducible placement on the patient are important. The 3D coils also ensure perfect fit to the body parts with complex geometries, like a neck, which is challenging to achieve with flexible 2D coils. To demonstrate how custom 3D printed coils can improve clinical imaging, we manufactured a custom neck array for c-spine and carotid artery imaging. Commercial neck coils are positioned at a distance from the body (Fig. S3a) to have large field of view and fit the majority of subjects, at the expense of SNR. High-resolution neck imaging is an indispensable tool for the evaluation of health conditions involving the neck and cervical spine. For instance, lesions^27–29^ or plaque accumulation in the carotid artery leading to stroke^28,30,31^ could be imaged and detected.

We used conventional and printed coil arrays to image a neck-shaped loading phantom and compared the SNR between the two coil arrays. The SNR measured with the printed array exceeded that of the commercially available four channel neck array (Siemens, Erlangen. Shown in Fig. 3f) by forty percent in the center of the phantom and up to five hundred percent near the surface. Furthermore, we imaged a volunteer to generate high-resolution images of the neck and cervical spine. Images taken with the printed array exhibited less graininess and sharper tissue boundaries when compared to the images taken with the commercially available control and the same pulse sequence. Our fabrication approach for custom MRI coils can enhance high quality clinical or research subject imaging by ensuring an optimal fit of the MRI receive coils to body parts with complex geometries.

## Results

### Fabrication process for custom MRI receive coils

**Figure 1:**
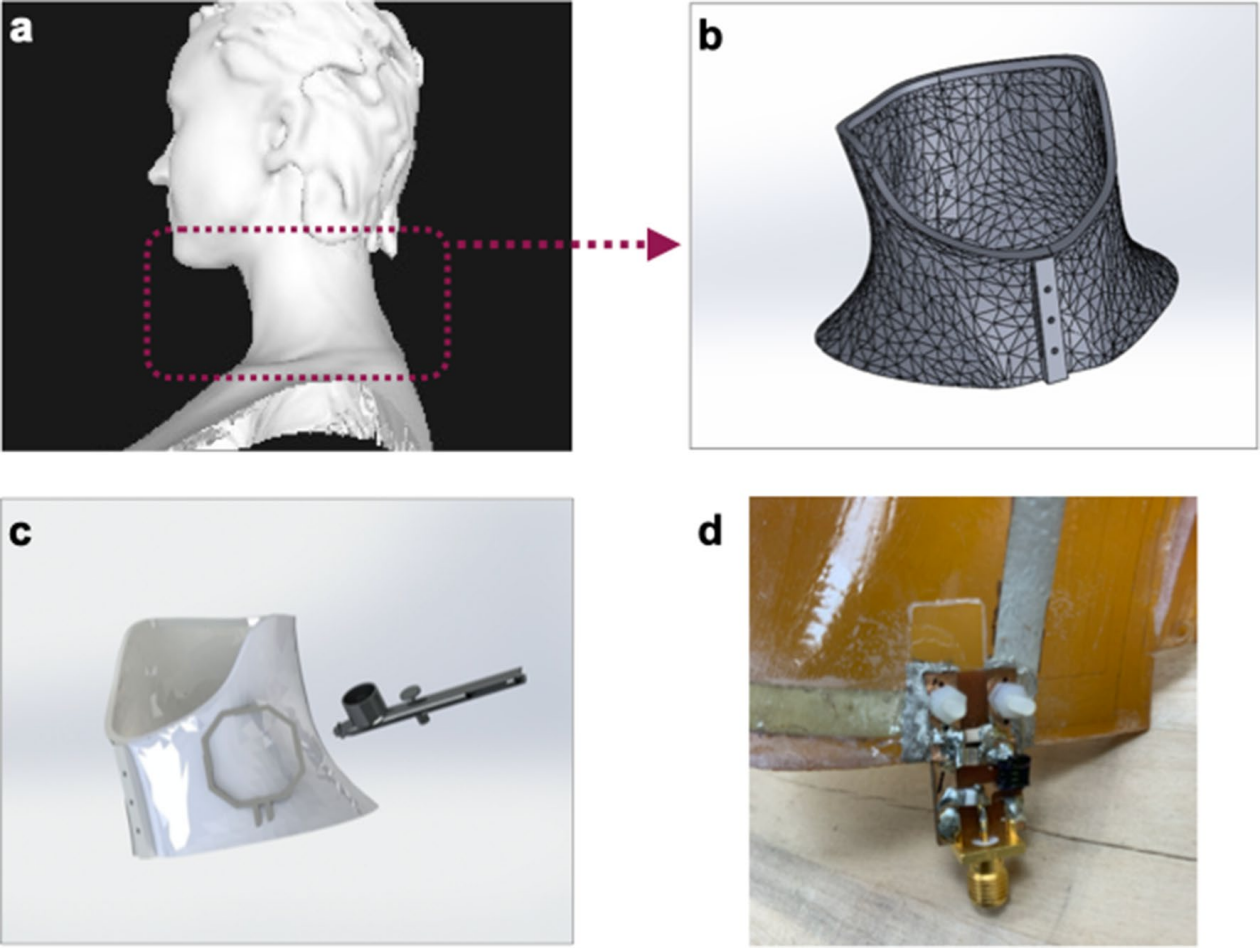
Manufacturing flow diagram for custom specific MRI receive cells. (**a**) A scan of the volunteer’s body part (neck) generated using a structural scanner. (**b**) CAD drawing generated using the structural scan to perfectly fit the volunteer’s neck and used to 3D-print a custom substrate. (**c**) Schematic representation of spray deposing coil components onto the 3D-printed custom substrate. (**d**) Q spoiling and matching circuitry connected to the coil with plastic screws and conductive epoxy.

The process for fabricating patient-specific MRI coils was designed to minimize the number of steps to enable on-demand manufacturing to drastically reduce the production lead time. In the first step, the patient’s body part of interest is scanned using a commercially available hand-held structure sensor (Fig. 1a). Then, a custom substrate of that region is 3D printed (Fig. 1b). Finally, the solution-processed electronic materials are spray deposited onto these substrates to form the conductors and capacitors of the MRI receive coils (Fig. 1c). This workflow can be readily adapted to the number of existing automated additive manufacturing approaches. We used the Carbon (Redwood City, CA) 3D printing process to fabricate the custom substrate. It enables the use of MRI transparent, heat and flame-resistant materials, as well as the ability to print monolithic parts with minimal artifacts of print anisotropy. We chose spray-deposition to layer coil components onto the substrate as it allows for rapid deposition of a wide range of electronic materials onto curvilinear surfaces. In order to spray-deposit patterns of desired geometry and eliminate the need for custom tooling or molds, custom masks were designed and 3D printed along with the substrate (Fig. S1). Alternative deposition techniques such as aerosol jet or extrusion printing could be used in place of spraying to eliminate the need for printed masks.

### Characterization and optimization of the coil components

**Figure 2:**
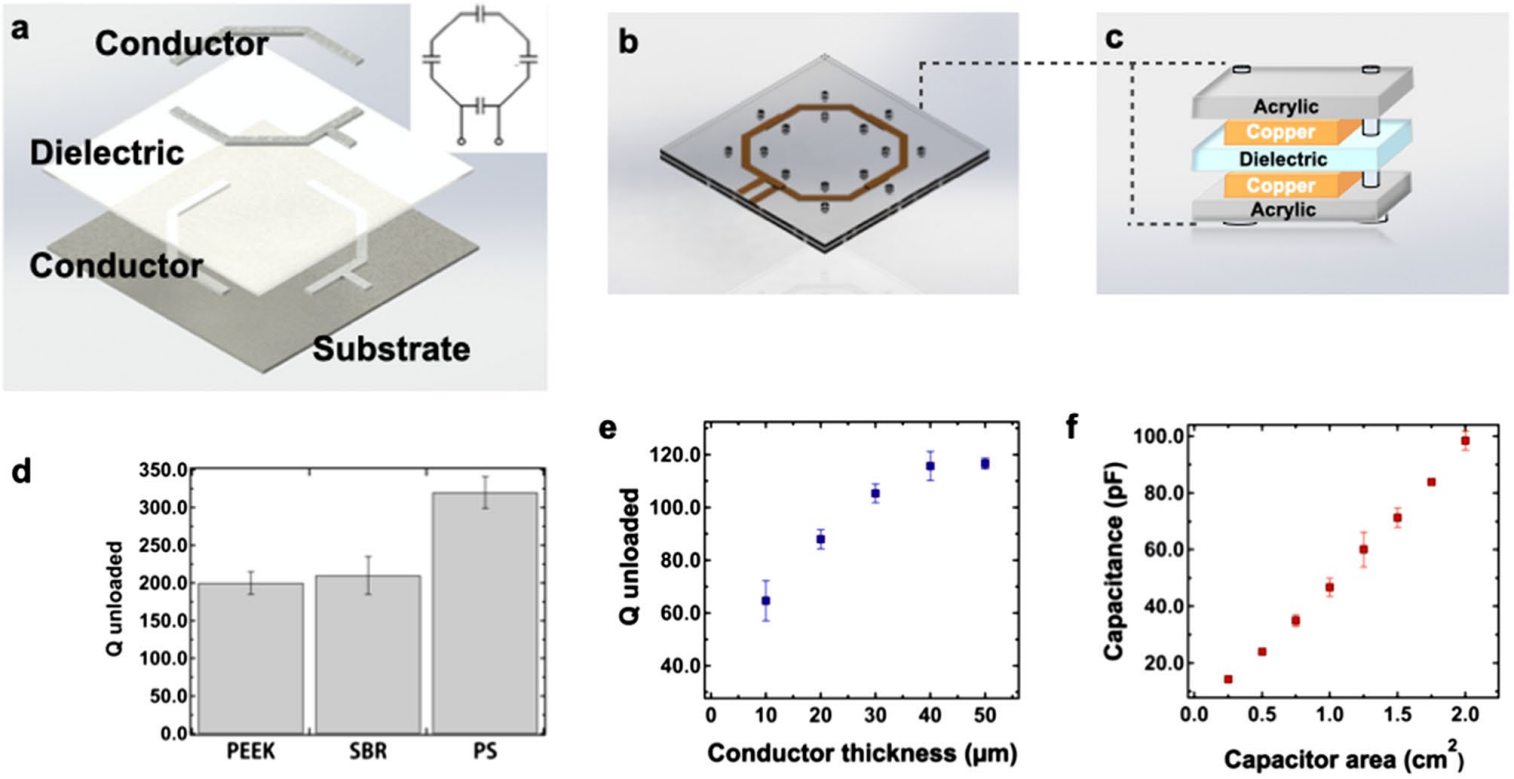
Characterization and optimization of coil components. (**a**) Schematic of the coil components that are sequentially deposited into the substrate. The conductor traces are designed to overlap, forming coils with capacitors evenly spaced throughout the loop. Schematics of the top view (**b**) and cross section (**c**) of the custom setup used to characterize dielectric properties of the candidate materials. Dielectric films are spray-deposited onto the copper traces fabricated from the commercial foil. The copper coils are clamped together with the dielectric in between using two acrylic sheets. (**d**) Q unloaded of the styrene butadiene resin (SBR) and polystyrene (PS) dielectric films sprayed from the solution, and commercially available polyether ether ketone (PEEK) film. (**e**) Effect of conductor thickness on the Q unloaded. (**f**) Dependence of capacitance on the top electrode area.

Conventional MRI coils are resonant LC circuits formed with a loop of conductive wire and rigid porcelain capacitors. In order to achieve such a circuit through spray deposition, we adapted a coil design previously reported by Corea et al. comprised of two silver conductor patterns with a dielectric layer separating them in certain areas^9^. Figure 2a shows the schematics of the corresponding layers that are sequentially deposited onto the substrate. The conductor traces were designed to overlap, forming coils with four parallel plate capacitors evenly spaced throughout the loop.

Careful selection of materials is essential for achieving high performing sprayed MRI coils. Losses determined by the coil materials and fabrication approach can be characterized by measuring the quality factor in the absence of a sample (Q unloaded), which is inversely related to the loss contribution from the coil^10^. Losses from both dielectric and conductor material can contribute to a decrease in Q unloaded and, consequently, compromise SNR. Additionally, materials used to fabricate MRI receive coils must be MR Safe, as defined in the American Society for Testing and Materials (ASTM) standard F2503-05. Materials should also be tested for MR transparency by following the guidelines described in ASTM F2119. Furthermore, the substrate used in the current process must withstand exposure to high temperatures during the annealing process of the silver conductor. With all considerations, we selected a cyanate ester resin material from Carbon to fabricate the substrate. Cyanate ester’s mechanical properties such as tensile strength and heat deflection temperature exceed those of commonly used plastics for MR devices like ABS and polycarbonate. Using alternative curing approaches such as pulse forge (Novacentrix) or UV curing would eliminate the need for heat resistive substrates and expand the pool of suitable substrate materials.

The dielectric was chosen based on its ability to be spray-deposited from a solution, glass transition temperature of over 100 °C to withstand the silver curing, and low dielectric loss to form high quality printed capacitors. Dielectric losses were measured at 123.3 MHz, the characteristic frequency of our 3 T MRI system. To characterize dielectric properties of candidate materials, we measured Q unloaded using a setup shown in Fig. 2b. In this setup, a 60 μm thick dielectric film was spray-deposited onto 70 μm thick copper traces cut from a commercially available foil. Since the commercial copper foil is highly conductive (resistivity = 1.68 × 10^−6^ Ω-cm), losses of such coils are dominated by the dielectric loss of the tested films and the measured value of Q unloaded can serve an indicator of the dielectric properties of the films. The copper coils were tuned to a resonance frequency of 123.3 MHz and were clamped around the dielectric midlayer using two acrylic sheets (Fig. 2c). Q unloaded was then measured using a network analyzer centered at the Larmor frequency (123.3 MHz). This testing setup has previously been shown to produce reliable Q unloaded measurements of dielectric films with minimum interference from the acrylic sheets^13^.

Figure 2d compares the unloaded Q of spray-deposited styrene butadiene rubber (SBR) and polystyrene (PS) dielectric films to the unloaded Q of a commercially available polyether ether ketone (PEEK) film previously used to fabricate printed flexible MRI coils. Measured values were averaged over three trials to equal 200, 210 and 320 for PEEK, SBR and PS respectively. The Q unloaded value of the PEEK film was consistent with the previously reported results^10^, confirming the reliability of the testing setup. The PS film had the highest Q unloaded and was chosen as the dielectric for the custom MRI coils. We further verified that dielectric properties of the PS films would not be affected by heating during silver curing. We exposed the films to the temperatures ranging from 25 to 110 °C for 30 min (the amount of time required to cure the silver layer, Fig. S2a). The resulting average Q fluctuated between 318 and 341 (Fig. S2b) without direct correlation to temperature. Such fluctuations could be partially attributed to the variations in positioning and orientation of the setup that affect the measured Q on a network analyzer.

A commercially available silver ink (PSPI 0250, Novacentrix) was used to create the conductive layers. It is an aqueous nanoparticle ink formulated for spray deposition processes. The water-based ink does not solvate the PS layer which helps to avoid short-circuiting between the top and bottom conductor traces. This ink can also be cured at relatively low temperatures of 80–100 °C, which is below the glass transition temperature of polystyrene (Fig. S2a). Fully printed coils fabricated with 10 μm thick printed conductor traces and a PS dielectric layer (Fig. S2c) resulted in a measured Q unloaded of 65 compared to the Q of 320 obtained with copper foil traces. This indicated that losses in the coil were dominated by the conductivity of the printed silver film. Printing additional silver layers increases the thickness and the conductivity of the silver traces and, in turn, the Q unloaded of the coil. We found that Q unloaded increases to the maximum value of ~ 120 when the thickness of the conductor reaches 40 μm (Fig. 2e). Further increases in conductor thickness has negligible effect on Q unloaded, as the conductivity of materials in AC current is limited by the skin depth effect. Therefore, we printed 40-μm-thick silver traces to fabricate all subsequent coils.

It is important to note that in the clinical settings, imaging losses from the patient dominate the overall loss of the system^18,19^, and after a certain point, an increase in Q unloaded does not meaningfully improve the SNR. Our group previously demonstrated a method relating Q unloaded of the printed coils to its SNR in an image of a homogeneous phantom mimicking human tissue^10^. The results showed that increasing Q unloaded above 100 would only increase SNR of the printed coil by ~ 3% compared to the control coil consisting of metal copper traces with porcelain capacitors. Thus, although the Q unloaded of the fully printed coils is lower compared to the Q of the coils with the copper foil traces, the difference between the two is negligible for all practical purposes. In contrast, the ability to place custom coils close to the body would result in significant SNR gains. The distance between a custom coil and patient is set by the thickness of the substrate to be on the order of ~ 0.5 cm, eliminating the loss in SNR and minimizing the capacitive coupling to the patient.

Changing the inductance and capacitance controls the resonant frequency of the coil. In the current process, the size and geometry of the loop fixes the inductance. We therefore adjust the capacitance to tune the coil to the Larmor frequency. Figure 2f demonstrates that varying area of a capacitor from 0.25 to 2 cm^2^ results in capacitance values ranging from 13 to 102 pF, which is sufficient to reach specific frequencies used in the MRI scanner (123.3 MHz; Siemens 3 T Trio, Erlangen, Germany).

### SNR from the custom coil array

Our approach aims to enhance high quality clinical imaging by ensuring an optimal fit of the MRI receive coils to the body parts. In order to demonstrate the benefits of using custom coils in clinical setting, we designed a custom array to image the neck and compared the SNR between conventional and printed coil arrays.

**Figure 3:**
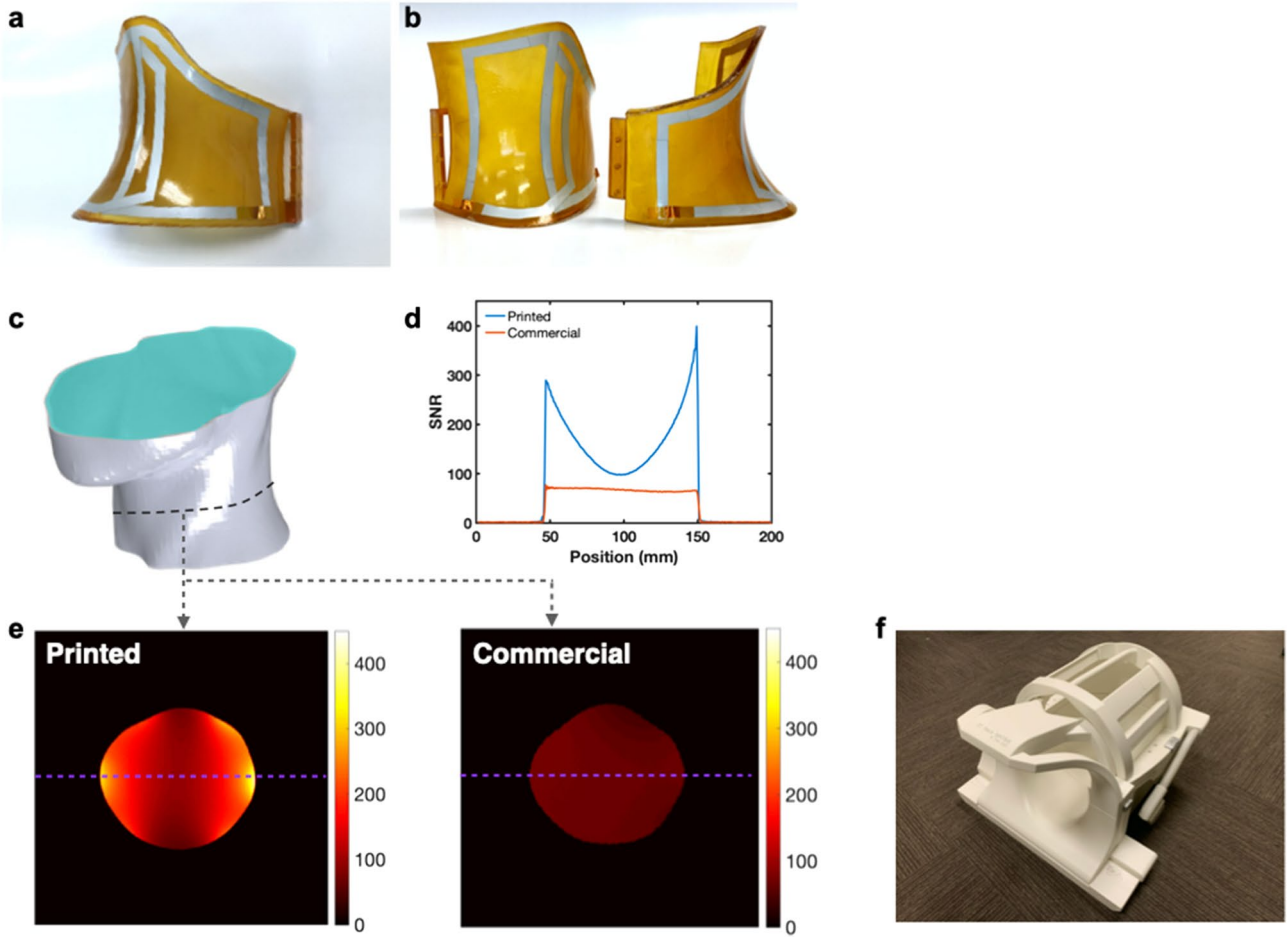
SNR from the custom coil array. (**a**) Photograph of the custom array fabricated by printing a pair of two-coil elements onto the custom substrate. (**b**) Photograph of the top custom arrays covering the entire neck surface area. (**c**) Schematics of the phantom consisting of 3D printed casing in a shape of the patient’s neck and containing solution of salts mimicking human tissue. (**d**) SNR of the printed and commercial coils along the cross-section of the slice through the middle of the neck. (**e**) SNR maps of the phantom using the printed and commercial arrays. Dashed lines indicate the location of SNR cross sections shown in (**d**). SNR was normalized to the maximum seen with the printed array. (**f**) Image of the commercially available 4 channel neck coil attached to a 12 channel head coil.

The custom array was fabricated by printing a pair of two-coil elements onto the substrate covering the entire neck surface area for a total of four channels (Fig. 3a,b). The elements’ shape was adjusted to fit the substrate and maximize the coverage, while retaining the original four-capacitor circuit design. Neighboring coils were overlapped to minimize coupling. The commercially available neck array (Siemens Healthineers, Erlangen) consisted of four elements operating in an integrated fashion with the head matrix coil (Fig. S3a). Both arrays were used to image a homogeneous loading phantom consisting of 3D printed casing in a shape of the patient’s neck and containing solution of salts mimicking human tissue (Fig. 3c).

Due to improved conformability to the phantom and the higher number of coil elements, the printed array produced higher SNR throughout the cross section of the phantom. Figure 3d compares SNR of the printed and commercial coils along the cross section of a slice though the middle of the neck. The SNR of the printed array exceeds that of the commercial array by forty percent near the center of the phantom and up to five hundred percent near the surface. The effect of coil proximity on the SNR can also be clearly seen from the SNR maps shown in Fig. 3e, where the regions near the conductive traces of the elements yielded the highest signal. Thus, custom printed coil arrays can yield higher SNR compared to commercially available counterparts, despite the lower performance characteristics of the solution-processed materials used for printing of the coil components. This translates into higher quality images taken by the custom array, having less graininess and more clearly differentiated tissue interfaces (Fig. S4). The experiment demonstrates that high quality custom 3D printed and sprayed coils are possible.

### In-vivo imaging

**Figure 4:**
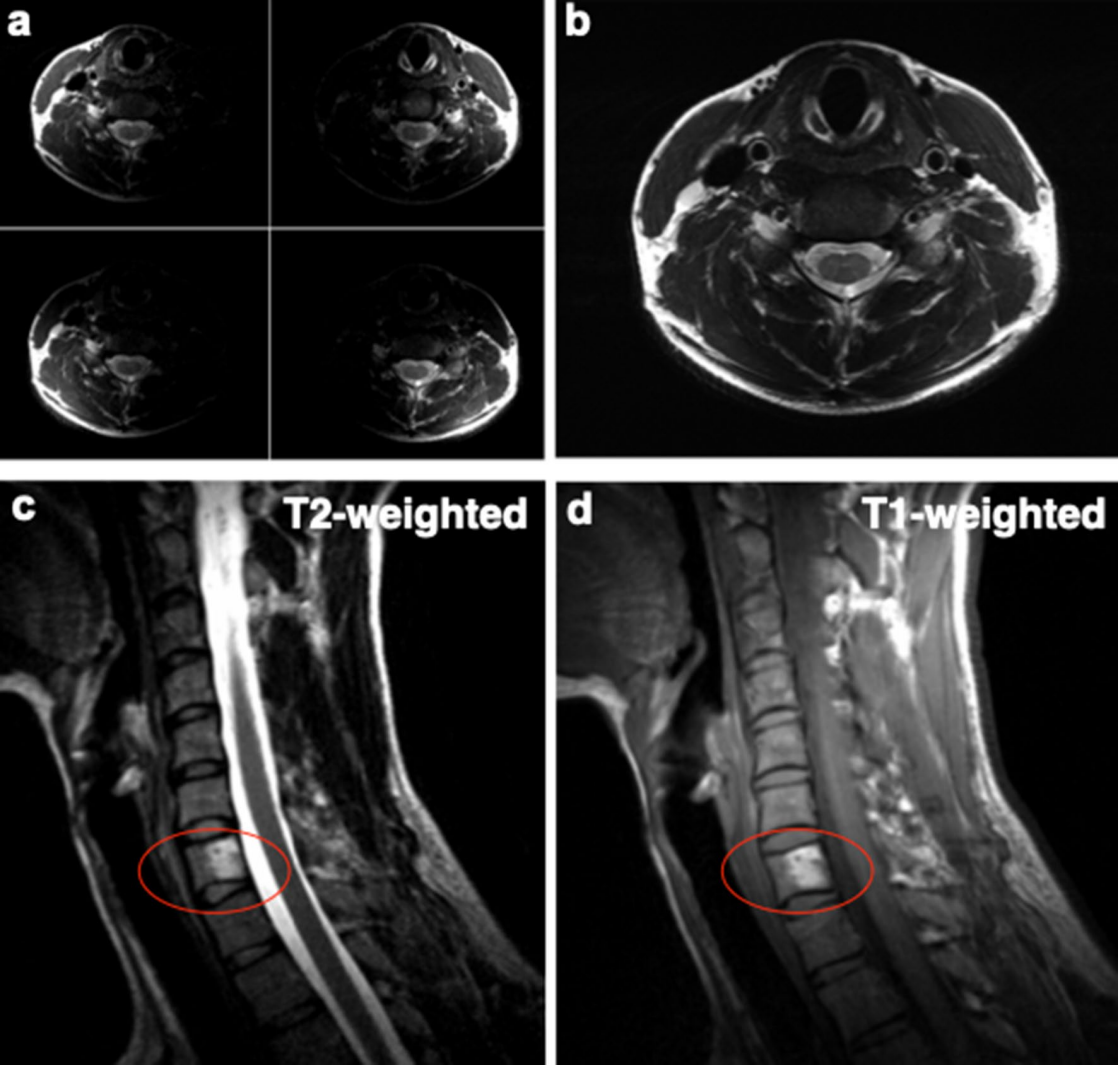
Imaging of the body parts relevant for clinical applications. (**a**) Images of the neck cross-section taken by individual elements. (**b**) Combined image of the neck cross-section. T2 (**c**) and T1 (**d**) weighted sagittal images of the spine of the volunteer with vertebral hemangioma (indicated with red line).

We used the 3D printed MRI coils described above for imaging the neck of a volunteer to demonstrate how they could be used in a clinical setting. The scanning was performed at UC Berkeley following an Institutional Review Board (IRB) approved protocol (2013-07-5491). Prior to the scan, informed consent was obtained from the subject for their participation. The subject also granted permission to publish data and photos from the scan. Figure 4a,b shows images of the neck cross-section taken by individual elements (a), as well as combined image (b). Each of the four elements contributes evenly to create a high-resolution image, with clearly differentiated anatomical features. Figure 4c,d shows the sagittal image of the spine of the volunteer with an incidental finding of vertebral hemangioma, a benign vascular tumor consisting primarily of blood vessels and fat tissue. T2-weighted images were acquired with a turbo spin echo (TSE) sequence with a repetition time (TR) of 3500 ms, and an echo time (TE) of 104 ms (Fig. 4c). T1-weighted images were acquired with a TSE sequence with a TR of 700 ms and TE of 10 ms (Fig. 4d). The short TR and TE result in an intrinsic T1 weighting since spins with a longer T1 do not fully recover before the next RF pulse. Different tissues are characterized by different intensity of T1 and T2 weighted images. Fat tissue shows increased signal intensity on T1-weighted images, while blood appears brighter on T2-weighted images. Since hemangioma has high content of both fat and blood—it appears bright on both T1 and T2-weighted images, as shown in Fig. 4c,d.

## Discussion

We present the first 3D MRI patient specific sprayed coils fabricated via additive manufacturing approaches. In spite of the higher resistivity and dielectric loss of solution-processed materials, custom printed coil arrays that conformed to the neck yielded higher SNR than a commercially available coil array. This resulted in images with less graininess and more clearly differentiated tissue interfaces. A limitation of this study is that the comparison is done with non-conforming coils. While other commercial coils exist, we did not have access to them. We attribute most of the SNR gain to the proximity of the custom coil to the phantom and its smaller element size. In addition, some SNR differences could be caused by the fact that the commercial array was not tuned and matched specifically for the phantom that we used. Another possible limitation is that the effects of B1 were not compensated for in the SNR measurements. However, we did not notice significant intensity changes in body coil images taken with the custom array placed on the phantom, hence any apparent SNR difference due to B1 would be small. In addition, the blocking impedance of the q-spoiling circuits was greater than 400 ohms for all channels. For our coil area of 64 cm^2^ the recommended blocking impedance to produce less than 1% level of artifacts is 358 ohms according to Kocharian et al.^34^. The SNR image (Fig. 3e) has inhomogeneities at the edges due to the high signal at the proximity of each pair of surface coils. Wang et al. report that SNR drops off as a cubic function of distance from the coil^36^. SNR is also lower at the top and bottom since the sprayed coils do not cover those regions to allow the coil to split in half, as illustrated in Fig. 3b.

Despite the ability to easily deposit conductors and dielectric materials to form the coil elements, fine tuning and matching is still a laborious process. One benefit of the sprayed coil is that the capacitance can be adjusted by scraping away portions of the silver traces. This method is simpler and easier than repeatedly reworking fixed capacitors by soldering.

The 3D custom coils could bring substantial value in applications where in addition to higher SNR and reproducible placement on the patient is important, such as MRI guided surgeries. Though our prototype coil was designed as a proof of concept for carotid imaging, we want to emphasize that our techniques could be extended to other body parts. The work presented here is a step towards a future where coil manufacturing could be fully automated. In addition, coils made with our methods could be designed with additional features to attach the coils to the scanner and restrict subject motion. At the same time, utilization of 3D printing and spray-deposition to fabricate the coil components allows rapid manufacturing of such coils. This manufacturing flow can be adapted to other automated deposition approaches such as extrusion or aerosol printing, further advancing widespread adoption of the approach.

## Methods

### Coil fabrication

Coils were produced by sequentially spray-depositing coil components onto a 3D printed substrate. The substrate of cyanate ester resin (Carbon) was printed along with masks defining the geometry of the conductor traces (Fig. S1) using a digital light synthesis (DLS) 3D printer (Carbon). The conductive traces were produced by spray-depositing an aqueous silver conductive ink (Novacentrix PSPI 0250/1000) onto a 3D printed substrate in the MRI coil trace pattern using a 3D printed mask. Subsequently, the metal layer was annealed at 100 °C for 30 min. The dielectric layer was then deposited onto the substrate, evenly coating the entire surface. Finally, the surface metal layer was spray deposited on top of the dielectric layer with an alternate 3D printed mask. Each sprayed silver layer took approximately five minutes to apply by hand. The dielectric layer was applied with an automated spray coating machine for about 3 h.

To fabricate a custom array, a pair of two-coil elements was printed onto the substrate covering the entire neck surface area. Rigid printed circuit boards (PCB) with Q-spoiling circuitry were attached to each coil with plastic screws (McMaster-Carr) as shown in Fig. 1d. An electrical connection was formed by direct contact between the copper on the PCB and the silver traces. The Q-spoiling circuit consisted of an inductor and PIN diode. When biased, the diode creates a resonant loop between the matching capacitor and the inductor creating a high impedance at the port of the coil^7,32^. This is used to detune the coil and protect the receiver circuitry during the transmit phase of an MR sequence. A three-quarter wavelength non-magnetic RG316 cable (Alpha Wire) with a BNC connector (Amphenol) was soldered directly to the PCB. The cost of the four-channel array (without the preamplifiers) was approximately $200, including the 3D printed substrate and rigid components.

### Characterization of the coil components

Dielectric properties of PS, SBR and PEEK were characterized by measuring Q unloaded of the coils fabricated with the corresponding dielectric as described below. PEEK film was purchased from Professional Plastics, PS and SBR films were deposited from solution. A 4% weight polystyrene (Sigma Aldrich, MW ~ 200,000 by GPC) solution in toluene (Sigma Aldrich, anhydrous 99.8%) was used to deposit polystyrene dielectric film. Premade aqueous SBR solution (EQ-Lib-SBR) was purchased from MTI corporation for making SBR dielectric. The corresponding solution was deposited onto 70 μm-thick copper foils cut into the shape of the MRI traces using a custom-built three axis stage fitted with an airbrush (Badger 350-4) set at 15 psi. The airbrush was repeatedly passed over the copper patterns, until 60 μm thickness of dielectric was deposited. During SBR deposition process copper traces were attached to a hot plate to maintain a surface temperature of 100 °C. Polystyrene films were deposited onto the surface at room temperature. The resulting film thicknesses were measured with a micrometer (Mitutoyo) after evaporating any remaining solvent (12hrs at room temperature for the polystyrene, 30 min at 100 °C for SBR). To assemble the complete coil, another bare copper foil trace was placed over the aforementioned traces, creating a closed inductive loop with a total of 4 capacitors. The completed coil was then sandwiched between two sheets of MRI transparent acrylic to regulate pressure on each capacitor across different tests.

To determine the optimum curing time and temperature for the silver nanoparticle ink (Fig. S2a) 0.5 cm by 4 cm strips of the ink were sprayed onto a PET substrate until a film of 40 μm was deposited. A four-point probe was used to measure the initial resistivity of the samples in three different positions 1 cm apart from each other. The samples were then dried at 60 °C, 80 °C and 100 °C with the resistivity measured periodically until no change was observed.

The conductor thickness was optimized by fabricating coils with varying silver trace thicknesses on planar substrates (Fig. S2c). The unloaded Q was measured for each sample and the silver thickness was chosen based on the point of diminishing returns due to the skin depth effect of alternating currents.

### Coil characterization

All coils were tested with an Agilent E5061B ENA network analyzer. To measure Q unloaded, each coil was calibrated to the correct resonance frequency of 123.3 MHz by varying capacitor areas. Capacitor area was changed by gently removing material from the top conductive trace. Two broadband loops were placed 30 cm apart to minimize the S21 noise floor. Then, the coil was placed in between the probes. Extra caution was taken to remove any conductive material from the testing zone to avoid risk of unintentionally loading the coil. Unloaded Q was measured from the S21 response by dividing the center frequency by the − 3 dB bandwidth at a span of 25 MHz. Coils were matched to an input impedance of 50 ohms by adjusting the value of the matching capacitor while measuring S11 on a loading phantom. Preamp decoupling was tuned by adjusting the length of the coaxial cable by small increments while the preamps were powered and connected to the coil. Three inductively coupled cable traps were attached to each cable to reduce common mode currents on the shield of the coax.

### Imaging

The SNR of the custom array was compared to that of the commercial array by imaging a conductive phantom on a 3 T scanner (Siemens 3 T Trio). The phantom consisted of the 3D printed casing in the shape of the patient’s neck, containing a solution of 3.356 g l^−1^ NiCl_2_ ∙ 6H_2_O and 2.4 g l^−1^ NaCl for conductivity of 0.68 Sm^−1^ at 123–127 MHz.

Each coil was connected to a 4-channel interface box (Stark Contrast, Erlangen, Germany) containing low noise preamplifiers. The custom coil array was stress tested with a one hour long, high SAR turbo spin echo pulse sequence. After the scan, the entire apparatus was removed from the scanner and immediately imaged with a thermal camera (FLIR Systems, Wilsonville OR) to ensure that the temperature of components that might contact a subject did not rise more than 15 °C above ambient, as required by our IRB approved protocol.

SNR maps were derived from a 2D gradient echo sequence with an echo time (TE) of 10 ms, repetition time (TR) of 438 ms, a flip angle of 25°, a resolution of 0.8 × 0.8 × 5 mm^3^, and a bandwidth of 260 Hz/pixel. Noise scans were acquired by running the same scan with the transmit voltage set to 0 V. Image analysis was conducted on the raw data files from the scanner. SNR maps were calculated in absolute units with methods described by Kellman et al.^35^ using noise pre-whitening and optimal coil combination.

To scan a volunteer the custom array was placed on the neck. Axial and sagittal T1 weighted images of the neck were taken with a turbo spin echo sequence (TE: 10 ms, TR: 700 ms, ETL: 5, Res: 0.6 × 0.8 × 3 mm^3^) with an inherent T1 weighting due to the short TR. In addition, axial and sagittal T2 weighted images of the neck were acquired with a turbo spin echo sequence (TE: 104 ms, TR: 3500 ms, ETL: 5, Res: 0.6 × 0.8 × 3 mm^3^).

### Ethics statement

All methods were performed in accordance with the relevant guidelines and regulations. Experiments on human subjects were carried out with informed consent under the approval of the UC Berkeley Committee for Protection of Human Subjects (CPHS) protocol 2013-07-5491. Prior to the experiment, our participant was screened for any MRI specific contraindications such as metal implants or pregnancy. The subject was advised about the risks of Magnetic Resonance Imaging and given the opportunity to stop the exam at any time. In addition, the subject provided informed consent to publish data and photographs from the experiment.

## Supplementary Information

Supplementary Figure S1.

Supplementary Figure S2.

Supplementary Figure S3.

Supplementary Figure S4.
