# Supplementary figures and images for: Custom, spray coated receive coils for magnetic resonance imaging
Source: Sci Rep. 2021 Jan 29;11:2635. doi: 10.1038/s41598-021-81833-0 (PMC7846777; doi:10.1038/s41598-021-81833-0)

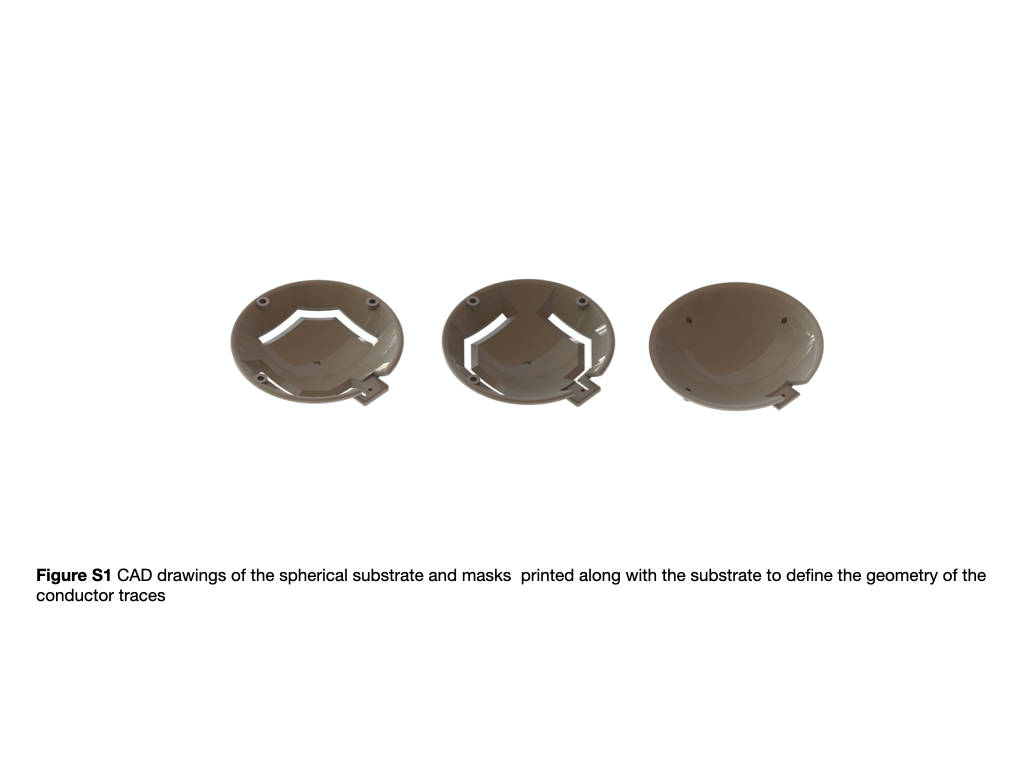

Supplement: Supplementary file 1 — Supplementary Figure S1. [file 41598_2021_81833_MOESM1_ESM.tiff]

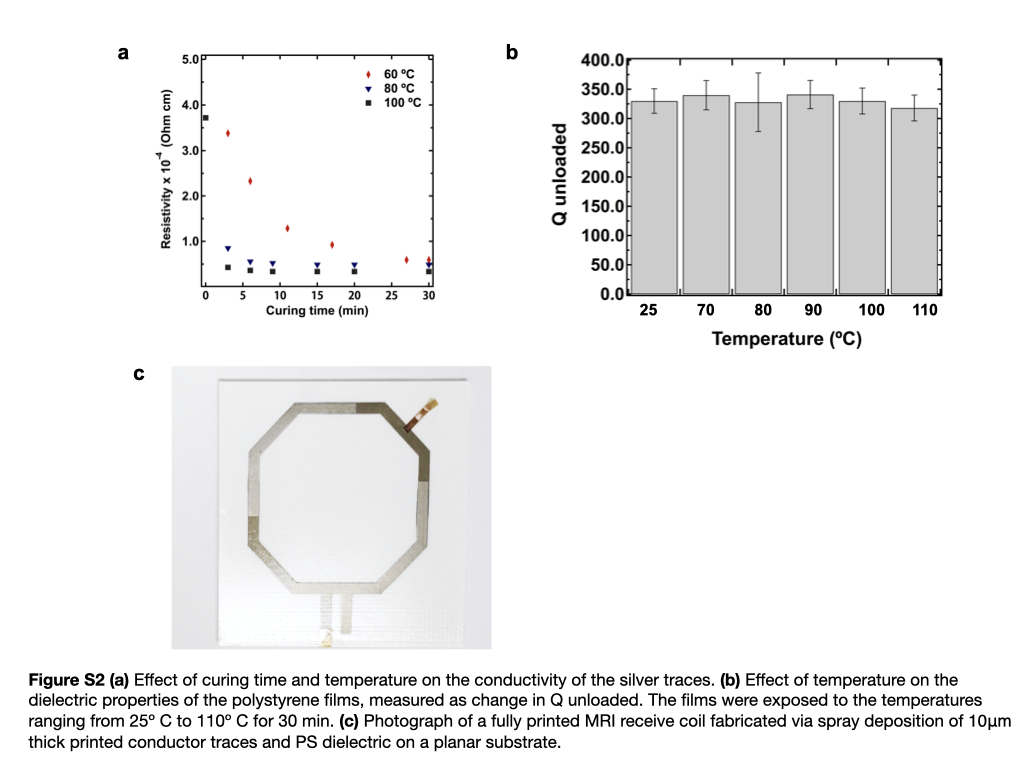

Supplement: Supplementary file 2 — Supplementary Figure S2. [file 41598_2021_81833_MOESM2_ESM.tiff]

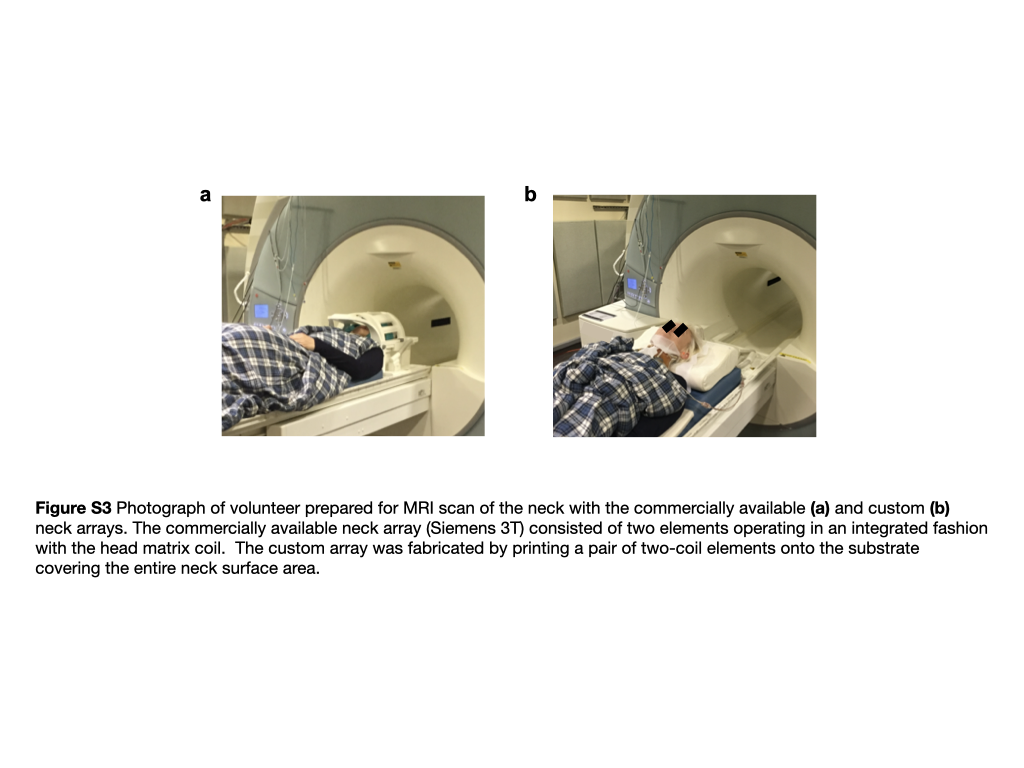

Supplement: Supplementary file 3 — Supplementary Figure S3. [file 41598_2021_81833_MOESM3_ESM.tiff]

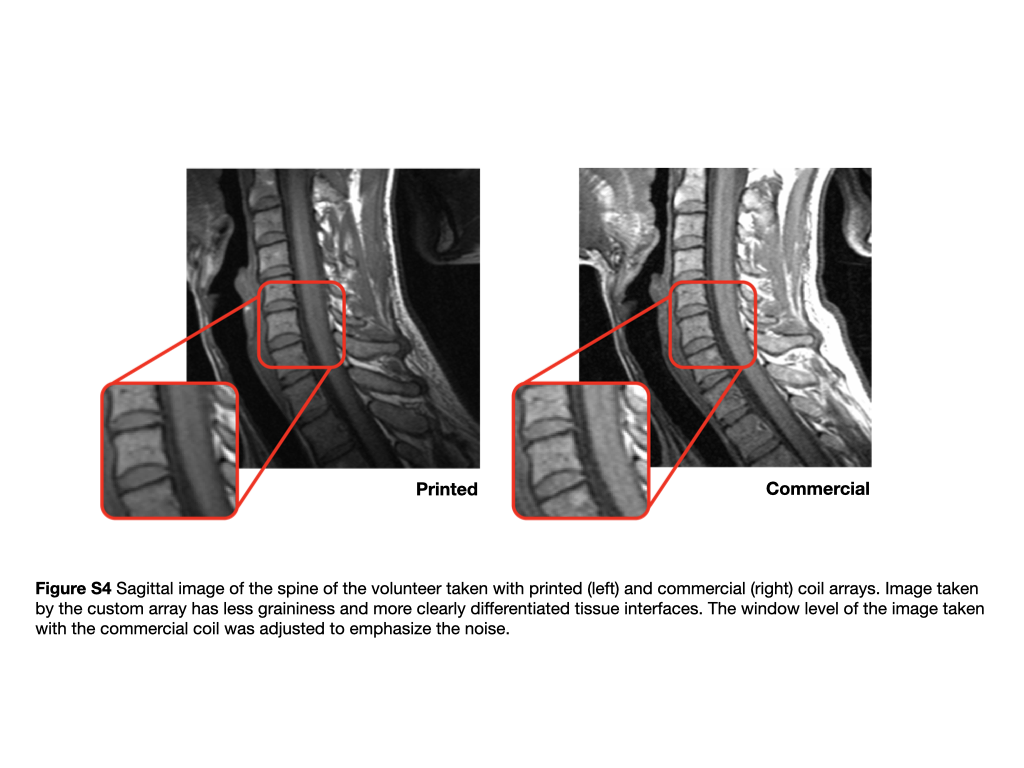

Supplement: Supplementary file 4 — Supplementary Figure S4. [file 41598_2021_81833_MOESM4_ESM.tiff]
